# Prognostic significance of lymphocyte PD-1 expression in combination with clinical scoring systems in patients with liver cirrhosis complicated by sepsis

**DOI:** 10.3389/fimmu.2026.1749708

**Published:** 2026-05-13

**Authors:** Zhao Liu, Fengwei Shi, Nan Geng, Wen Pan, Bo Liu, Qinghua Meng

**Affiliations:** 1 Department of Emergency Medicine, Beijing Youan Hospital, Capital Medical University, Beijing, China; 2 Beijing Institute of Hepatology, Beijing Youan Hospital, Capital Medical University, Beijing, China; 3 Department of Respiratory and Critical Care Medicine, Beijing Youan Hospital, Capital Medical University, Beijing, China; 4 Department of Liver Disease, Beijing Youan Hospital, Capital Medical University, Beijing, China

**Keywords:** clinical scoring systems, liver cirrhosis, PD-1 expression, prognosis, sepsis

## Abstract

**Background:**

Liver cirrhosis complicated by sepsis is associated with significant immune dysfunction and high mortality rates. Programmed cell death protein 1 (PD-1) expression on lymphocytes has been implicated in immune exhaustion, but its prognostic significance in this population remains unclear.

**Objective:**

This study aimed to evaluate the prognostic value of lymphocyte PD-1 expression in combination with clinical scoring systems, such as the Child-Pugh score and chronic liver failure-sequential organ failure assessment (CLIF-SOFA), for predicting disease severity and 28-day mortality in patients with cirrhosis complicated by sepsis.

**Methods:**

This prospective study included 86 patients with cirrhosis and sepsis admitted to Beijing You’an Hospital between June 2023 and May 1, 2024. Fresh whole blood was collected at enrollment, and peripheral blood mononuclear cells (PBMCs) were isolated for flow cytometric analysis of PD-1 expression on CD3+, CD4+, and CD8+ lymphocytes. Clinical parameters, scoring systems, and laboratory data were collected to assess their correlation with PD-1 expression and patient outcomes.

**Results:**

Of the 86 patients, 38 (44.2%) survived, while 48 (55.8%) died within 28 days. Non-survivors exhibited higher PD-1 expression on CD3+, CD4+, and CD8+ lymphocytes, as well as worse Child-Pugh and CLIF-SOFA scores (all p < 0.05). A significant positive correlation was observed between lymphocyte PD-1 expression and disease severity scores. Multivariate logistic regression analysis identified PD-1 expression on CD3+, CD4+, and CD8+ lymphocytes as independent predictors of 28-day mortality. Combining lymphocyte PD-1 expression with clinical scoring systems improved the predictive accuracy for septic shock and mortality.

**Conclusion:**

Lymphocyte PD-1 expression is associated with disease severity and poor prognosis in patients with cirrhosis complicated by sepsis. Combining PD-1 expression with traditional scoring systems enhances risk stratification and may provide a more comprehensive tool for predicting outcomes in this high-risk population.

## Introduction

Liver cirrhosis is a chronic and progressive condition characterized by extensive hepatic fibrosis and impaired liver function, often leading to severe complications, including sepsis (1). Sepsis, a life-threatening organ dysfunction caused by a dysregulated host immune response to infection, is a major cause of morbidity and mortality in patients with advanced liver disease (2). The coexistence of cirrhosis and sepsis poses significant challenges to clinicians due to the complex interplay between immune dysfunction, systemic inflammation, and organ failure (3, 4). Despite advancements in critical care, predicting the severity and outcomes of cirrhosis complicated by sepsis remains difficult, highlighting the need for more effective prognostic tools (5–7).

Programmed cell death protein 1 (PD-1), an immune checkpoint receptor expressed on activated T cells, plays a critical role in immune regulation (8, 9). Excessive PD-1 expression on lymphocytes in sepsis reflects an immune exhaustion state, characterized by reduced T-cell proliferation, cytokine production, and impaired immune response to infections (10). This overexpression leads to a compromised ability to clear pathogens effectively, increasing susceptibility to secondary infections and persistent inflammation (11). Studies have shown that higher levels of PD-1 expression on lymphocytes are associated with greater disease severity, multi-organ failure, and higher mortality rates in septic patients (12–14). In patients with liver cirrhosis, lymphocyte function is often impaired due to chronic immune activation and systemic inflammation, resulting in immune exhaustion and reduced pathogen clearance (15–17), this is similar to sepsis (18).

However, the prognostic significance of lymphocyte PD-1 expression in patients with cirrhosis complicated by sepsis has not been fully explored. Additionally, clinical scoring systems such as the Child-Pugh score and CLIF-SOFA score are widely used to assess disease severity in patients with liver cirrhosis and sepsis (19–23), but their predictive accuracy may be limited when used alone.

This study aimed to investigate the prognostic value of lymphocyte PD-1 expression in combination with clinical scoring systems for predicting disease severity and 28-day mortality in patients with liver cirrhosis complicated by sepsis. By evaluating the correlation between lymphocyte PD-1 expression, clinical parameters, and scoring systems, we sought to provide a more comprehensive approach to risk stratification and outcome prediction in this high-risk population. Our findings offer novel insights into the role of immune biomarkers and their integration with traditional scoring systems to enhance prognostic accuracy.

## Patients and methods

### Study design and participants

We conducted a prospective study involving 86 patients with cirrhosis complicated by sepsis who were admitted to the emergency department of Beijing You’an Hospital between June 2023 and May 1, 2024. The study included baseline sampling at admission and a 28-day follow-up to assess all-cause mortality. The diagnosis of cirrhosis was based on the diagnostic criteria outlined in the 2019 edition of the Guidelines for the Diagnosis and Treatment of Cirrhosis issued by the Chinese Society of Hepatology (24). Sepsis was diagnosed according to the Sepsis-3 criteria, which define sepsis as life-threatening organ dysfunction caused by a dysregulated host response to infection (2). The diagnostic criteria for septic shock included the need for vasopressors to maintain a mean arterial pressure (MAP) ≥ 65 mmHg despite adequate fluid resuscitation, accompanied by a blood lactate level ≥ 2 mmol/L. The aim of the study was to investigate the value of lymphocyte PD-1 expression combined with clinical scoring systems in predicting disease severity and prognosis in patients with cirrhosis complicated by sepsis. We have also included data from the healthy population, as well as from patients with cirrhosis only and patients with sepsis only. The study was approved by the Ethics Committee of Beijing You’an Hospital, Capital Medical University (Approval No. LL-2023-006-K) and conducted in accordance with the principles of the Helsinki Declaration. Informed consent was obtained from all participants, and all data were anonymized to ensure privacy.

### Inclusion and exclusion criteria

Inclusion Criteria: Participants were eligible if they were aged 18 years or older, regardless of gender, and had a diagnosis of cirrhosis based on the 2019 edition of the Guidelines for the Diagnosis and Treatment of Cirrhosis issued by the Chinese Society of Hepatology. Additionally, they were required to meet the Sepsis-3 criteria for the diagnosis of sepsis proposed at the 2016 International Sepsis Conference and provide signed informed consent to participate in the study.

Exclusion Criteria: Exclusion criteria included patients who did not agree to participate, those with HIV infection, long-term use of immunosuppressants, thyroid dysfunction, or active pulmonary tuberculosis. Pregnant individuals, patients younger than 18 years, and those with missing important data were also excluded.

### Data collection

The data collected for this study included demographic information, primary liver disease, comorbidities, vital signs, disease severity scores, and laboratory parameters. Demographic data consisted of sex and age, while primary liver disease included etiologies such as hepatitis B viral hepatitis, hepatitis C viral hepatitis, alcoholic hepatitis, autoimmune hepatitis, and liver cancer. The presence of liver failure was also recorded. Comorbidities included hepatic encephalopathy, gastrointestinal bleeding, ascites, diabetes mellitus, coronary heart disease, pneumonia, chronic obstructive pulmonary disease (COPD), kidney failure, and other malignant tumors. Vital signs such as body temperature, respiratory rate (RR), heart rate (HR), and systolic blood pressure (SBP) were measured.

Disease severity was assessed using the Child-Pugh score and classification (Grades B and C) and the CLIF-SOFA score (19, 20, 23). Laboratory parameters included inflammatory markers such as procalcitonin (PCT) and C-reactive protein (CRP); hematological parameters such as hemoglobin (HGB), white blood cell (WBC) count, neutrophil count, lymphocyte count, and neutrophil-to-lymphocyte ratio (NLR); coagulation markers such as international normalized ratio (INR) and D-dimer; metabolic markers such as glucose and lactic acid; and liver function markers such as alanine aminotransferase (ALT), aspartate aminotransferase (AST), total bilirubin (TBIL), and direct bilirubin (DBIL). Immune parameters were also measured, including CD3+, CD4+, and CD8+ cells, as well as programmed cell death protein 1 (PD-1) expression on CD3+, CD4+, and CD8+ cells. This comprehensive dataset allowed for a detailed analysis of the clinical characteristics, disease severity, and immune status of patients with cirrhosis complicated by sepsis.

### Blood sample collection and testing

Fresh whole blood (10 mL) was collected from each patient and the healthy population at the time of enrollment using anticoagulant tubes. The blood samples were processed within four hours of collection to ensure optimal sample quality. Red blood cells were lysed using BD Pharm Lyse™ lysing buffer (Cat# 555899, BD Biosciences). Peripheral blood mononuclear cells (PBMCs) were then isolated using density gradient centrifugation. After separation, the PBMCs were washed and resuspended in phosphate-buffered saline (PBS) for further analysis.

Flow cytometry was performed to analyze lymphocyte subsets and PD-1 expression. Fluorescently labeled antibodies were used for staining, including anti-CD3 (PC5, Beckman Coulter, Cat# A07749), anti-CD4 (PerCP, BD Pharmingen™, Cat# 566924), anti-CD8 (APC, BD Pharmingen™, Cat# 561421), and anti-PD-1 (PE, BD Pharmingen™, Cat# 560795). The PBMCs were incubated with these antibodies in the dark at room temperature for 30 minutes. After staining, the cells were washed with PBS, resuspended in a fixation buffer, and analyzed using a flow cytometer. Data acquisition and analysis were performed with FlowJo software, and appropriate gating strategies were applied to identify CD3+, CD4+, and CD8+ T cells, as well as their PD-1 expression levels. This method ensured precise and reliable detection of lymphocyte subsets and immune markers. The process of flow cytometric analysis is shown in **Supplementary Figure S1**.

### Statistical analysis

The Shapiro-Wilk test was applied to evaluate the normality of continuous variables. For variables that followed a normal distribution, data were expressed as mean ± standard deviation (SD) and compared using the independent-samples Student’s t-test. For non-normally distributed variables, data were presented as median with interquartile range (IQR: Q1; Q3) and analyzed using the Mann-Whitney U test. Categorical variables were summarized as counts and percentages, and comparisons were performed using Pearson’s chi-square test or Fisher’s exact test, as appropriate. Multiple samples were compared using the Analysis of Variance (ANOVA). A P-value of less than 0.05 was considered statistically significant. Univariate and multivariate logistic regression analyses were conducted to identify potential risk factors affecting patient prognosis. Spearman’s rank correlation was employed to explore the relationship between lymphocyte PD-1 expression and clinical variables such as age, vital signs, and laboratory markers. Kaplan-Meier survival curves were used to assess the predictive value of parameters for 28-day mortality. The Receiver Operating Characteristic (ROC) curve was utilized to evaluate the predictive ability of parameters for assessing disease severity and prognosis in patients with cirrhosis complicated by sepsis. The optimal cut-off value was determined by identifying the point with the highest Youden index. Data analysis was carried out using SPSS software (version 22.0; IBM Corp.) and R software (version 4.2.1; R Foundation for Statistical Computing), while visualizations were generated with GraphPad Prism 9 (GraphPad Software Inc.).

## Results

### Baseline characteristics and clinical profiles at admission

**Figure 1:**
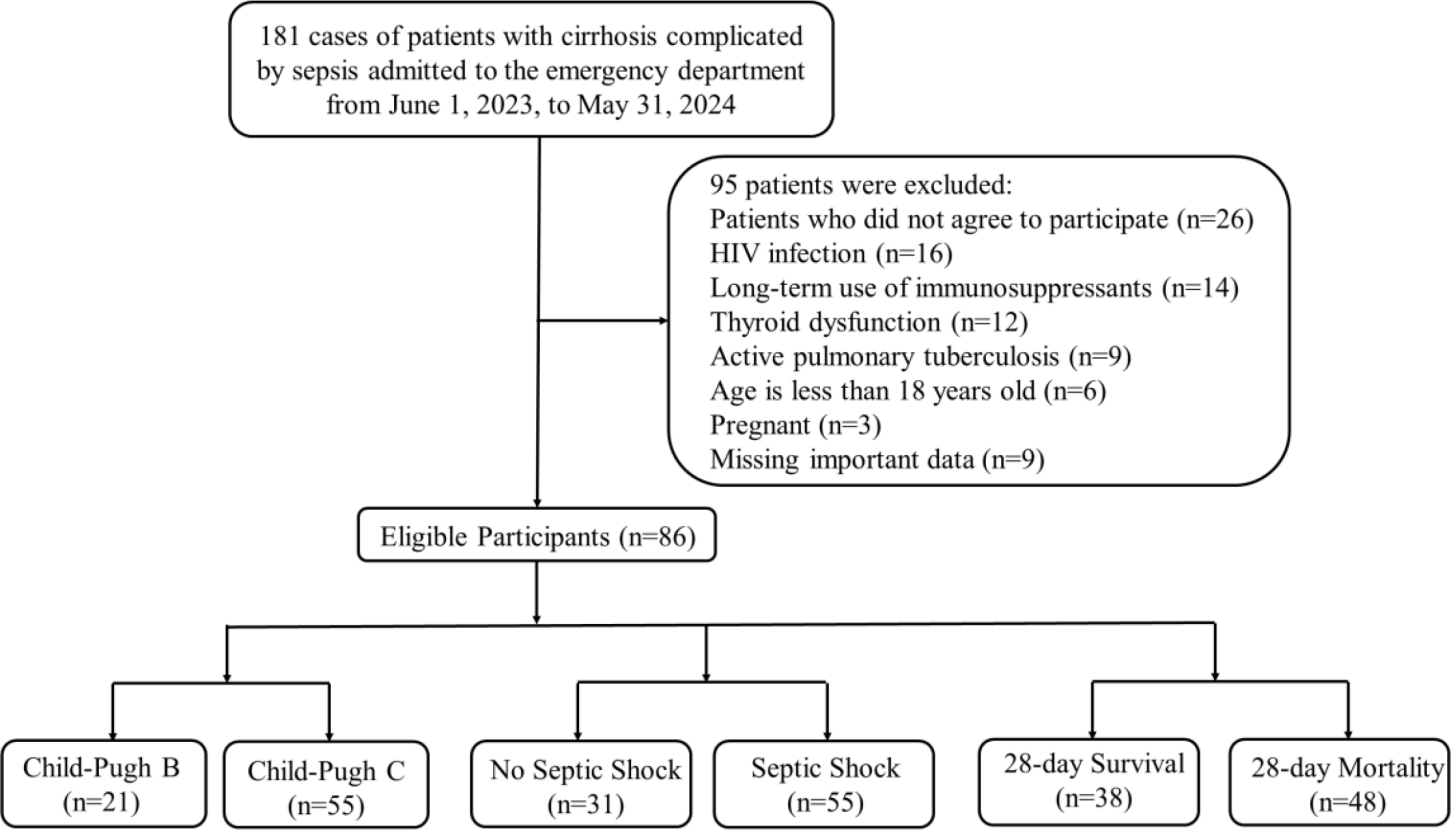
Flow diagram of patients enrollment.

A total of 181 patients were screened for eligibility, and 95 patients were excluded. Ultimately, 86 patients with cirrhosis complicated by sepsis were included in the final analysis. Based on the Child-Pugh classification, 21 patients (24.4%) were categorized as Grade B, while 55 patients (63.9%) were classified as Grade C. According to septic shock status, 31 patients (36.0%) did not present with shock, whereas 55 patients (64.0%) had septic shock. Regarding 28-day outcomes, 38 patients (44.2%) survived, while 48 patients (55.8%) died. Details are presented in **Figure 1**.

**Table 1** summarizes the baseline characteristics and clinical parameters of patients with cirrhosis complicated by sepsis. Of the 86 patients included in the study, 62 (72.1%) were male, and the median age was 60 years. The most common primary liver diseases were alcoholic hepatitis (26/86, 30.2%) and hepatitis B viral hepatitis (24/86, 27.9%). Additionally, 46 patients (53.5%) had liver failure.

**Table 1:** Baseline characteristics and clinical data after hospitalization of patients with cirrhosis complicated by sepsis.

Variables	Total (n=86)	28-day survival (n=38)	28-day mortality (n=48)	P-value
Demographic data				
Sex, male, n (%)	62 (72.1%)	29 (76.3%)	33 (68.8%)	0.593
Age (years)	60 (12)	59 (9)	60 (14)	0.697
Primary liver disease				
Hepatitis B viral hepatitis (N, %)	24 (27.9%)	10 (26.3%)	14 (29.2%)	0.960
Hepatitis C viral hepatitis (N, %)	4 (4.7%)	1 (2.6%)	3 (6.2%)	0.627
Alcoholic hepatitis (N, %)	26 (30.2%)	12 (31.6%)	14 (29.2%)	0.996
Autoimmune hepatitis (N, %)	3 (3.5%)	1 (2.6%)	2 (4.2%)	1.000
liver cancer (N, %)	25 (29.1%)	11 (28.9%)	14 (29.2%)	1.000
liver failure (N, %)	46 (53.5%)	15 (39.5%)	31 (64.6%)	0.036*
Co-morbidities				
Hepatic encephalopathy	53 (61.6%)	15 (39.5%)	38 (79.2%)	<0.001*
Gastrointestinal bleeding	19 (22.1%)	8 (21.1%)	11 (22.9%)	1.000
Ascites	71 (82.6%)	28 (73.7%)	43 (89.6%)	0.100
Diabetes mellitus, n (%)	16 (18.6%)	9 (23.7%)	7 (14.6%)	0.425
Coronary heart disease, n (%)	25 (29.1%)	5 (13.2%)	20 (41.7%)	0.008*
Pneumonia, n (%)	30 (34.9%)	9 (23.7%)	21 (43.8%)	0.087
COPD, n (%)	6 (7.0%)	0 (0.0%)	6 (12.5%)	0.032*
Kidney failure, n (%)	57 (66.3%)	15 (39.5%)	42 (87.5%)	<0.001*
Other malignant tumor, n (%)	13 (15.1%)	6 (15.8%)	7 (14.6%)	1.000
Vital signs	26 (14%)	22 (13%)	4 (17%)	0.581
Body temperature, °C	37 [36.8;38.4]	37 [36.8;38.0]	37 [36.8;38.5]	0.255
RR, breaths/min	22 [20;24]	20 [20;22]	23 [21;27]	<0.001*
HR, beats/min	103 (20)	97 (21)	108 (19)	0.016*
SBP, mmHg	99 [83;121]	111 [92;126]	85 [80;106]	<0.001*
Disease severity				
Child-Pugh score	12 [10;13]	10 [9;12]	13 [11;14]	<0.001*
Child-Pugh classification (N, %)				<0.001*
A	0 (0%)	/	/	
B	21 (24.4%)	17 (44.7%)	4 (8.3%)	
C	65 (75.6%)	21 (55.3%)	44 (91.7%)	
CLIF-SOFA	11.0 [6.0;13.0]	6.0 [5.0;11.0]	12.0 [10.0;14.2]	<0.001*
Laboratory parameters				
PCT, ng/ml	4.9 [1.5;11.6]	3.4 [1.5;8.1]	5.7 [1.4;15.9]	0.333
CRP, mg/L	68.3 [30.1;123.0]	63.4 [32.3;124.9]	75.5 [30.1;120.2]	1.000
HGB, g/L	93.6 (26.8)	89.9 (27.0)	96.4 (26.6)	0.271
WBC count, ×109/L	10.0 [6.0;16.1]	11.0 [4.9;17.6]	9.6 [6.5;15.5]	0.801
Neutrophils count, ×109/L	9.0 [5.1;14.7]	9.8 [3.7;15.5]	8.2 [5.7;13.7]	0.896
Lymphocytes count, ×109/L	0.6 [0.4;1.1]	0.6 [0.4;1.1]	0.5 [0.3;1.1]	0.307
NLR	13.6 [7.5;23.7]	12.7 [7.5;20.0]	16.9 [8.0;25.3]	0.198
INR	1.7 [1.4;2.3]	1.4 [1.3;1.8]	2.1 [1.5;2.4]	<0.001*
D-dimer, mg/L	25.6 [4.3;2514.0]	10.8 [3.6;996.2]	836.0 [7.8;4048.0]	0.018*
Glucose, mmol/L	6.5 [5.1;8.6]	7.8 [5.7;9.4]	5.7 [4.6;7.9]	0.013*
Lactic acid, (mmol/L)	3.5 [1.9;6.7]	2.0 [1.6;4.2]	4.4 [3.2;8.5]	<0.001*
ALT, U/L	35.0 [19.5;67.8]	36.0 [22.8;64.8]	34.0 [19.0;68.0]	0.761
AST, U/L	67.5 [42.2;119.2]	66.5 [43.5;118.8]	70.5 [40.5;116.2]	0.972
TBIL, μ mol/L	102.2 [42.6;269.2]	53.2 [37.5;291.2]	140.7 [57.5;260.7]	0.159
DBIL, μ mol/L	68.0 [26.7;207.5]	36.7 [21.9;251.9]	91.2 [41.9;199.0]	0.167
CD3+ Cells,/μL	360 [211;679]	40 [221;707]	310 [198;582]	0.400
CD4+ Cells,/μL	217.0 [114.0;434.0]	231.0 [148.2;455.0]	182.0 [96.5;405.5]	0.343
CD8+ Cells,/μL	104.0 [55.0;144.0]	123.0 [49.8;168.0]	94.0 [59.5;125.0]	0.283
PD-1(+) CD3+ Cells, (%)	61.7 (14.3)	54.2 (10.5)	67.7 (14.2)	<0.001*
PD-1(+) CD4+ Cells, (%)	53.7 (11.6)	47.8 (9.1)	58.4 (11.2)	<0.001*
PD-1(+) CD8+ Cells, (%)	65.8 (13.1)	58.4 (9.6)	71.6 (12.5)	<0.001*

Normally distributed continuous variables are displayed as mean ± SD, standard deviation and were compared using the independent-samples Student’s *t test*. Non-normally distributed continuous variables are displayed as a median with interquartile range [IQR: Q1-Q3] and were compared using the Mann–Whitney U test. Categorical variables are expressed as counts with percentages and were compared using Pearson’s chi-square or Fisher’s exact test. COPD, Chronic Obstructive Pulmonary Disease; RR, respiratory rate; HR, heart rate; SBP, systolic blood pressure; CLIF-SOFA, chronic liver failure-sequential organ failure assessment; PCT, Procalcitonin; CRP, C-reactive protein; HGB, Hemoglobin; WBC, White blood cell; NLR, Neutrophil-to-lymphocyte ratio; INR, International normalized ratio; ALT, Alanine aminotransferase; AST, Aspartate aminotransferase; TBIL, Total bilirubin; DBIL, Direct bilirubin. *p-value <0.05 was considered significant.

Vital signs and disease severity scores also differed significantly between the two groups. Non-survivors had higher respiratory and heart rates, lower systolic blood pressure, and worse Child-Pugh and CLIF-SOFA scores (all p<0.05). In terms of laboratory and immune parameters, non-survivors exhibited higher INR, glucose, lactic acid, D-dimer levels, and percentages of PD-1(+) CD3+, CD4+, and CD8+ cells (all p<0.05).

### Correlation of lymphocyte PD-1 expression with clinical parameters and disease severity

**Figure 2:**
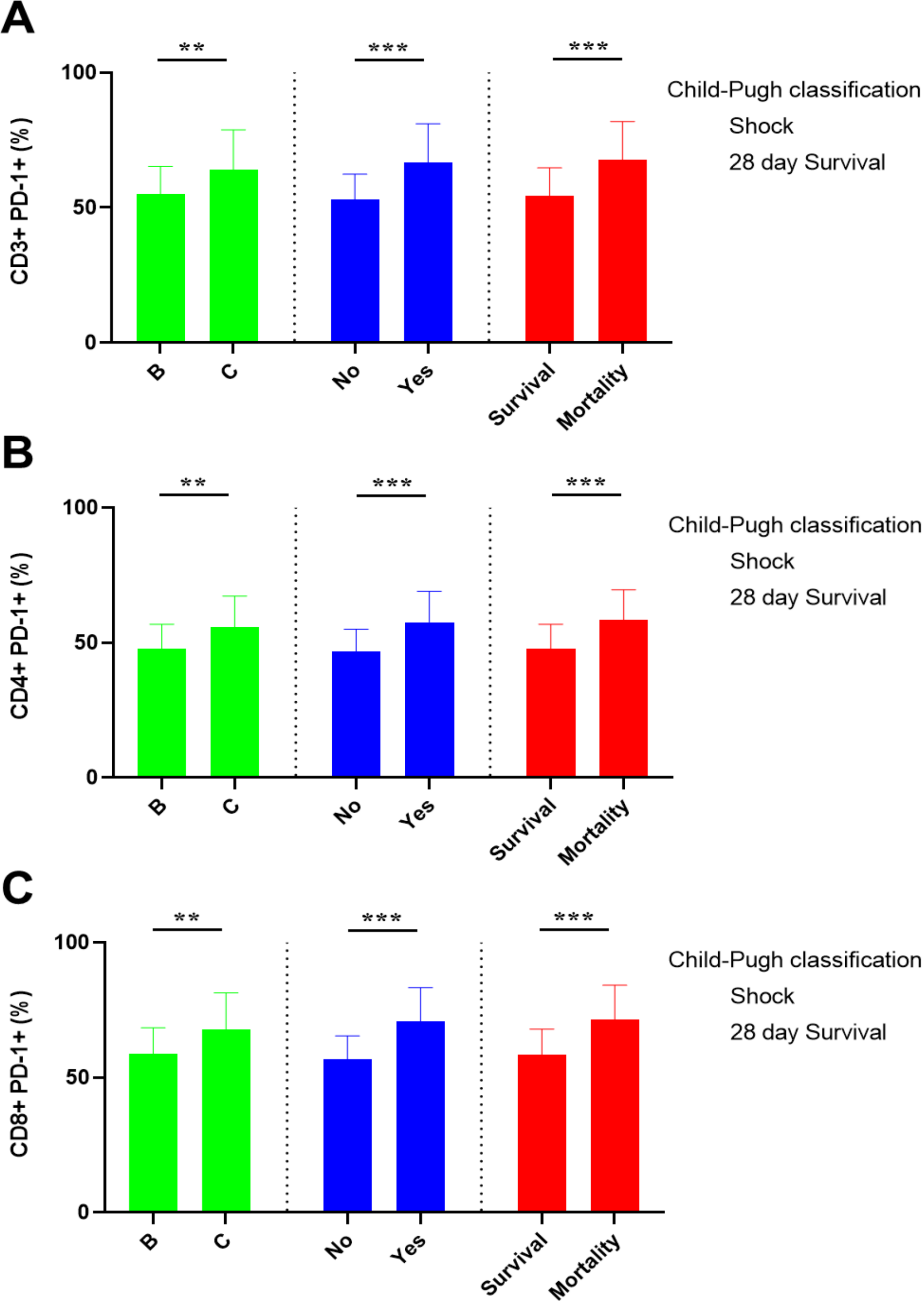
Lymphocyte PD-1 expression in patients with different disease severities and outcomes. This **Supplementary Figure** Shows the proportion of CD3+PD-1+ cells **(A)**, CD4+PD-1+ cells **(B)**, and CD8+PD-1+ cells **(C)** in patients categorized by disease severity and 28-day survival status. The data are displayed as mean ± SD, standard deviation and were compared using the independent-samples Student’s t-test. ** indicates p < 0.01, and *** indicates p < 0.001.

The lymphocyte PD-1 expression in different populations is shown in **Supplementary Figure S2**. In this study, all with cirrhosis complicated by sepsis were classified as Child-Pugh Grade B or C. lymphocyte PD-1 expression was higher in patients with Grade C compared to Grade B. Additionally, patients with septic shock exhibited higher levels of lymphocyte PD-1 expression compared to those without shock. Similarly, 28-day mortality patients showed higher lymphocyte PD-1 expression levels (**Figures 2A–C**).

**Figure 3:**
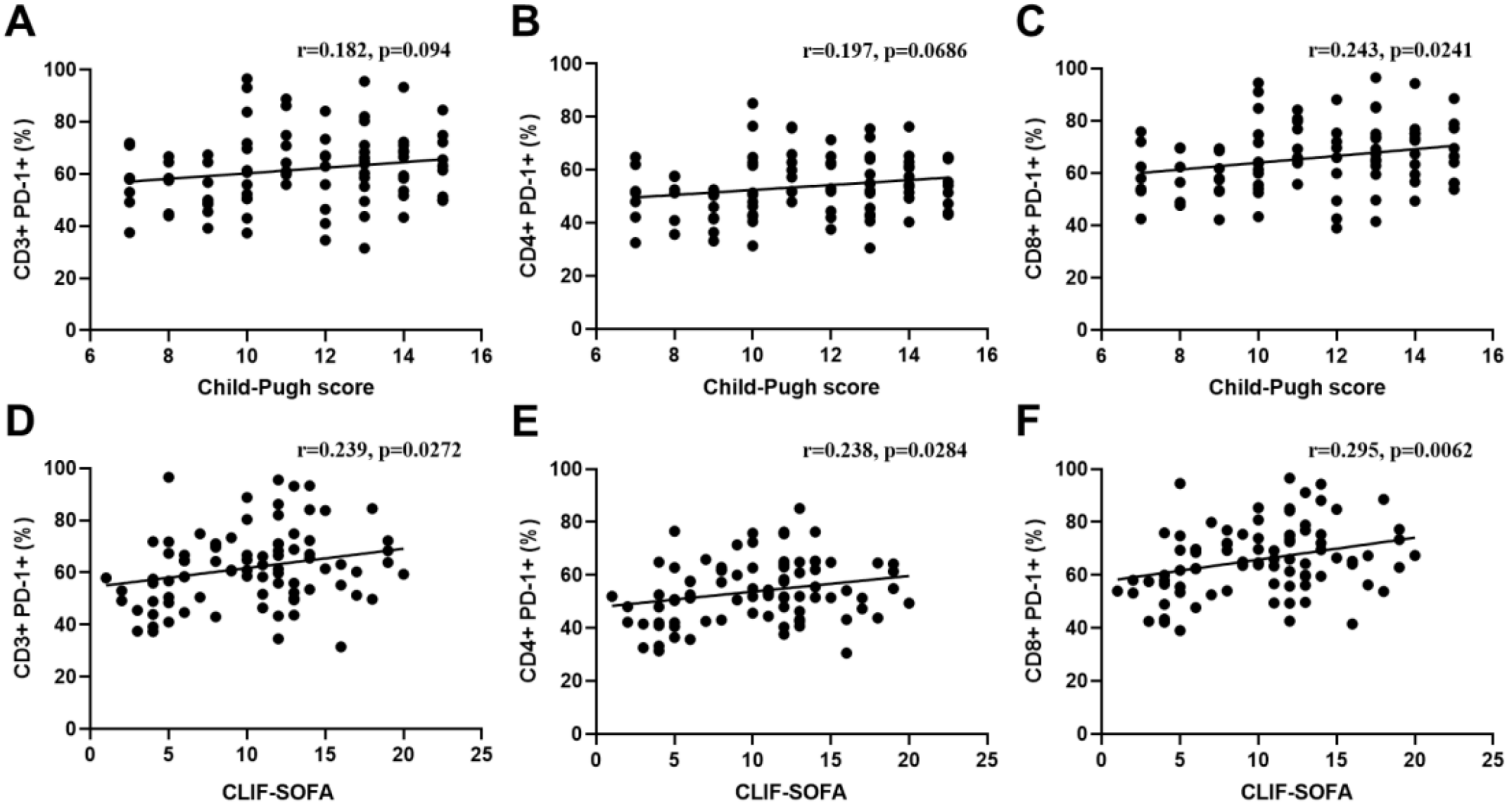
Correlation between lymphocyte PD-1 expression and clinical scores. **(A–C)** Correlations of CD3+PD-1+ cells (%), CD4+PD-1+ cells (%), and CD8+PD-1+ cells(%) with the Child-Pugh score, respectively. **(D–F)** Correlations of CD3+PD-1+ cells (%), CD4+PD-1+ cells (%), and CD8+PD-1+ cells (%) with the CLIF-SOFA score, respectively. Spearman’s rank correlation was used for the analysis, and p < 0.05 was considered statistically significant.

The correlation between Lymphocyte PD-1 Expression and Clinical Scores was also evaluated. A positive trend was observed between CD3+PD-1+ cells (%), CD4+PD-1+ cells (%), and the Child-Pugh score, but the differences were not statistically significant (**Figures 3A, B**). However, CD8+PD-1+ cells (%) showed a statistically significant positive correlation with the Child-Pugh score (p < 0.05) (**Figure 3C**). Additionally, CD3+PD-1+ cells (%), CD4+PD-1+ cells (%), and CD8+PD-1+ cells (%) were positively correlated with the CLIF-SOFA score, all with statistical significance (all p < 0.05), as shown in **Figures 3D–F**. The correlation between Lymphocyte PD-1 Expression and age, vital signs, and laboratory markers is displayed in the heatmap (**Supplementary Figure S3**).

### Lymphocyte PD-1 expression for predicting severity and prognosis in patients with cirrhosis complicated by sepsis

**Figure 4:**
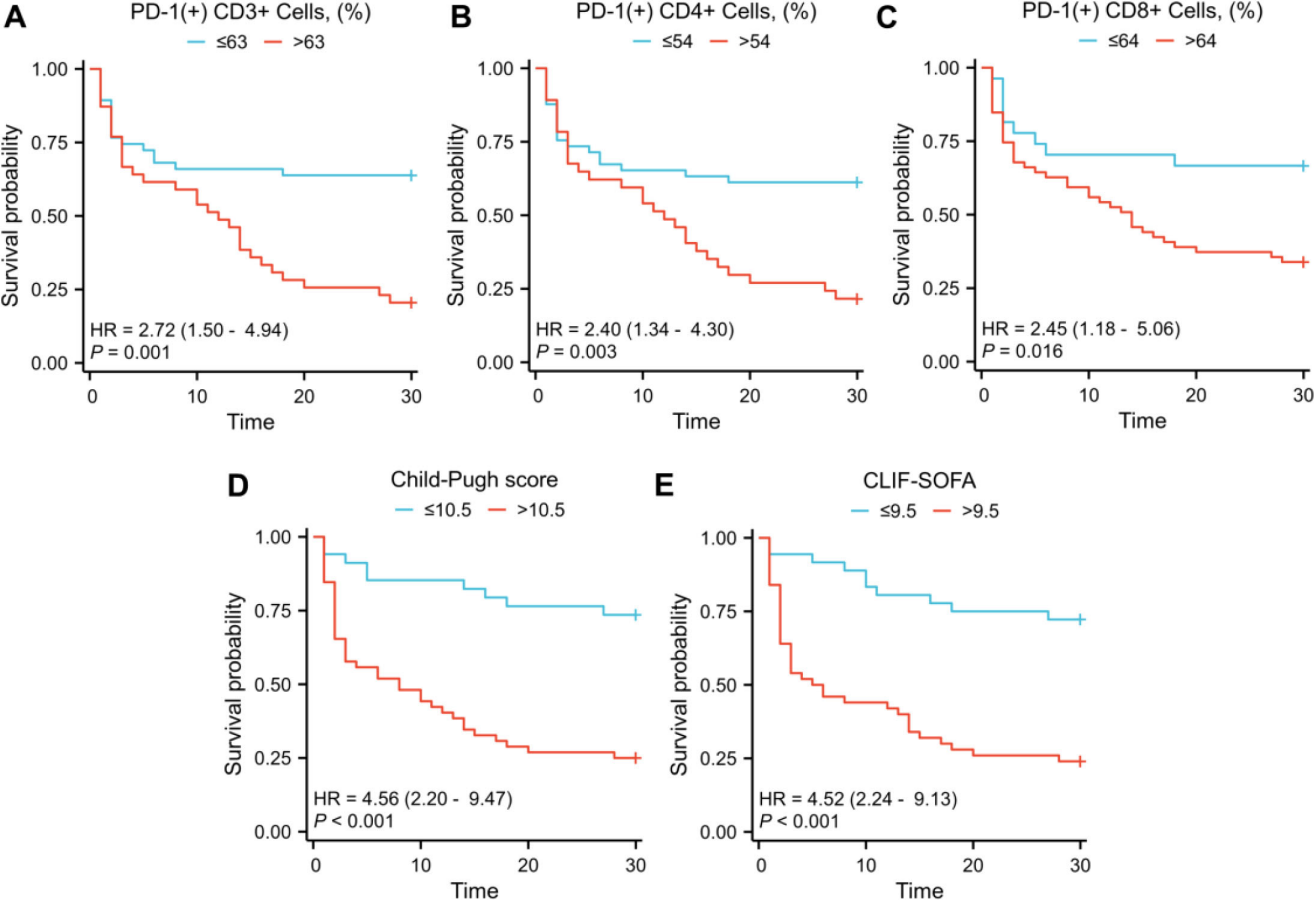
Kaplan-Meier curves for 28-day survival categorized by CD3+PD-1+ cells (%) **(A)**, CD4+PD-1+ cells (%) **(B)**, CD8+PD-1+ cells (%) **(C)**, Child-Pugh score **(D)** and CLIF-SOFA **(E)**. Abbreviations: CLIF-SOFA, chronic liver failure sequential organ failure assessment. p < 0.05 was considered statistically significant.

We performed univariate logistic regression analysis on the variables with statistical significance in **Table 1**. Variables with p < 0.1 were subsequently included in multivariate logistic regression analysis along with CD3+PD-1+ cells (%), CD4+PD-1+ cells (%), and CD8+PD-1+ cells (%). The results revealed that CD3+PD-1+ cells (%), CD4+PD-1+ cells (%), and CD8+PD-1+ cells (%) were independent predictors of 28-day mortality in patients with cirrhosis complicated by sepsis. Details are shown in **Supplementary Tables S1**–**S3** and **Table 2**. Survival curve analysis revealed that CD3+PD-1+ cells (%), CD4+PD-1+ cells (%), CD8+PD-1+ cells (%), Child-Pugh score, and CLIF-SOFA levels were associated with patient prognosis (**Figures 4A–E**).

**Table 2:** Univariable and multivariable logistic regression analysis for the predictors of 28-day mortality by clinical factors and PD-1(+) T lymphocytes.

Characteristics	Univariate analysis		Multivariate analysis	
	Odds ratio (95% CI)	P value	Odds ratio (95% CI)	P value
PD-1(+) CD3+ Cells, (%)	1.092 (1.045 – 1.143)	< 0.001*	1.144 (1.051 – 1.245)	0.002*
PD-1(+) CD4+ Cells, (%)	1.108 (1.051 – 1.167)	< 0.001*	1.158 (1.052 – 1.274)	0.003*
PD-1(+) CD8+ Cells, (%)	1.113 (1.057 – 1.171)	< 0.001*	1.160 (1.061 – 1.268)	0.001*

For variables with statistical differences in **Table 1**, univariate logistic regression analysis was conducted. Variables with a p-value less than 0.1 were included in the multivariable regression model for analysis with PD-1(+) CD3+ Cells, PD-1(+) CD4+ Cells, and PD-1(+) CD8+ Cells, respectively. The variables included liver failure, Hepatic encephalopathy, Coronary heart disease, COPD, Kidney failure, RR, HR, SBP, Child-Pugh score, CLIF-SOFA, INR, D-dimer, Glucose, and Lactic acid. For more details, refer to the **Supplementary Materials**, **Supplementary Tables S1**-**S3**. OR: odds ratio; 95% CI: 95% confidence interval. COPD, Chronic Obstructive Pulmonary Disease; RR, respiratory rate; HR, heart rate; SBP, systolic blood pressure; CLIF-SOFA, chronic liver failure-sequential organ failure assessment; INR, International normalized ratio. *p-value <0.05.

**Figure 5:**
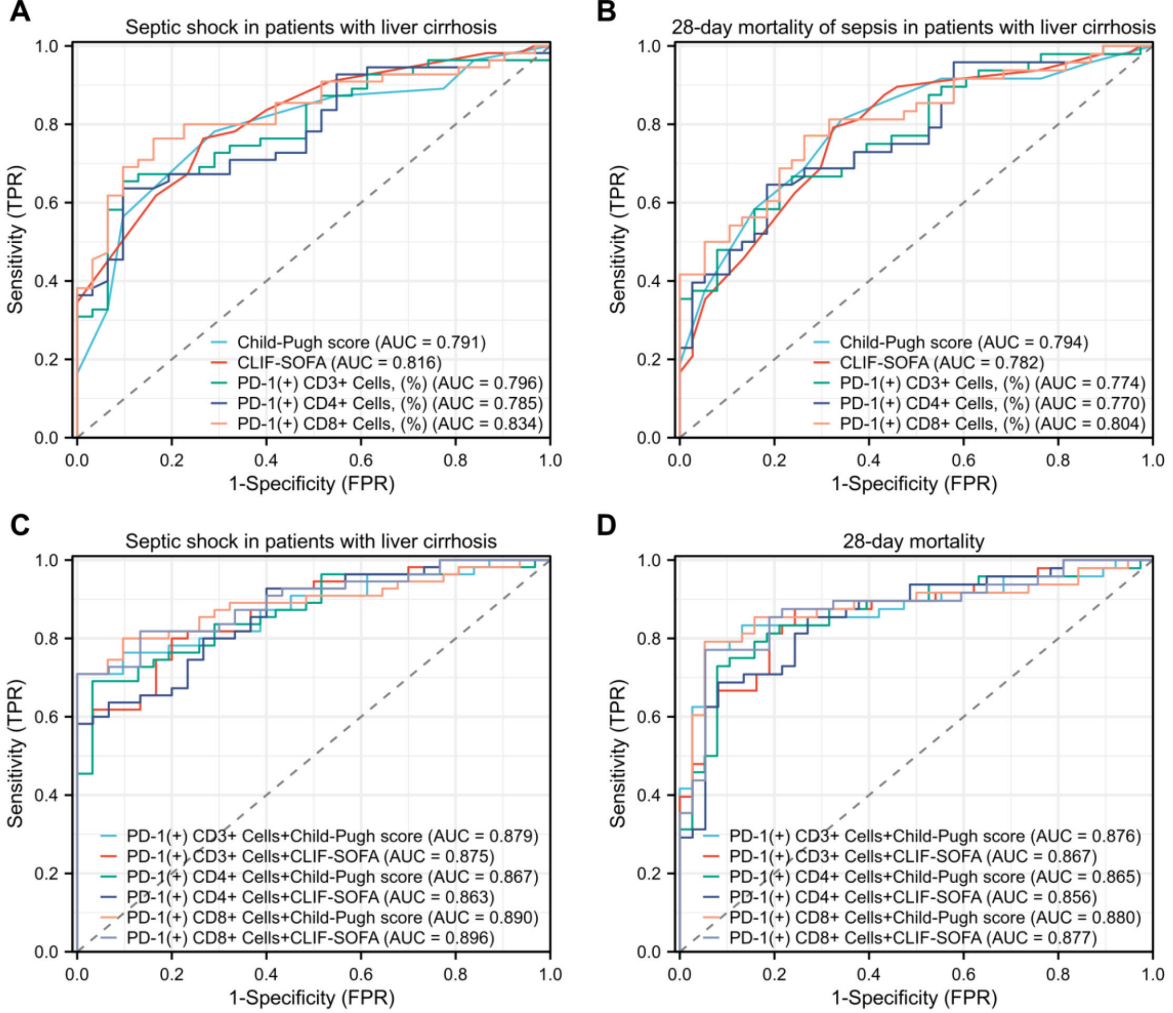
ROC curves of lymphocyte PD-1 expression and clinical scoring systems for severity and 28-day mortality of patients. **(A)** Predicting severity of patients. The AUC, area under the curve for Child-Pugh score was 0.791 (95% confidence interval [CI]: 0.694 – 0.888); CLIF-SOFA, AUC was 0.816 (95% CI: 0.726 – 0.906); CD3+PD-1+ cells (%), 0.796 (95% CI: 0.702 – 0.891); CD4+PD-1+ cells (%), AUC was 0.785 (95% CI: 0.689 – 0.881); CD8+PD-1+ cells (%), AUC was 0.834 (95% CI: 0.748 – 0.919). **(B)** Predicting 28-day mortality of patients. The AUC, area under the curve for Child-Pugh score was 0.794 (95% confidence interval [CI]: 0.699 – 0.892); CLIF-SOFA, AUC was 0.782 (95% CI: 0.684 – 0.880); CD3+PD-1+ cells (%), 0.774 (95% CI: 0.677 – 0.871); CD4+PD-1+ cells (%), AUC was 0.770 (95% CI: 0.672 – 0.868); CD8+PD-1+ cells(%), AUC was 0.804 (95% CI: 0.713 – 0.895). **(C)** Lymphocyte PD-1 expression combined with clinical scoring systems for predicting the severity of patients. The AUC, area under the curve for CD3+PD-1+ cells (%) combined with Child-Pugh score was 0.879 (95% confidence interval [CI]: 0.809 – 0.949); CD4+PD-1+ cells (%) combined with Child-Pugh score, AUC was 0.875 (95% CI: 0.804 – 0.946); CD8+PD-1+ cells (%) combined with Child-Pugh score, 0.867 (95% CI: 0.793 – 0.942); CD3+PD-1+ cells (%), AUC combined with CLIF-SOFA was 0.863 (95% confidence interval [CI]: 0.788 – 0.938); CD4+PD-1+ cells (%) combined with CLIF-SOFA, AUC was 0.890 (95% CI: 0.822 – 0.958); CD8+PD-1+ cells (%) combined with CLIF-SOFA, 0.896 (95% CI: 0.832 – 0.960). **(D)** Lymphocyte PD-1 expression combined with clinical scoring systems for predicting 28-day mortality of patients. The AUC, area under the curve for CD3+PD-1+ cells (%) combined with Child-Pugh score was 0.876 (95% confidence interval [CI]: 0.800 – 0.953); CD4+PD-1+ cells (%) combined with Child-Pugh score, AUC was 0.876 (95% CI: 0.791 – 0.943); CD8+PD-1+ cells (%) combined with Child-Pugh score, 0.865 (95% CI: 0.786 – 0.943); CD3+PD-1+ cells (%), AUC combined with CLIF-SOFA was 0.856 (95% confidence interval [CI]: 0.776 – 0.936); CD4+PD-1+ cells (%) combined with CLIF-SOFA, AUC was 0.880 (95% CI: 0.803 – 0.957); CD8+PD-1+ cells (%) combined with CLIF-SOFA, 0.877 (95% CI: 0.801 – 0.953).

Finally, we performed ROC curve analyses of lymphocyte PD-1 expression and clinical scoring systems for predicting the severity and 28-day mortality of patients. The results showed that lymphocyte PD-1 expression, Child-Pugh score, and CLIF-SOFA effectively predicted septic shock and 28-day mortality (**Figures 5A, B**). Moreover, combining lymphocyte PD-1 expression with the Child-Pugh score or CLIF-SOFA further improved the predictive value (**Figures 5C, D**). Detailed information is provided in **Table 3**.

**Table 3:** Predicted value information of clinical scores and PD-1(+) T cells for septic shock and prognosis in patients with liver cirrhosis.

Variables	Cut off value	Sensitivity	Specificity	PPV	NPV	Accuracy	Youden index
Predicting Septic shock							
Child-Pugh score	10.0	0.78	0.71	0.83	0.65	0.75	0.49
CLIF-SOFA	9.5	0.76	0.73	0.84	0.63	0.75	0.50
PD-1(+) CD3+ Cells, (%)	63.0	0.65	0.90	0.92	0.60	0.74	0.56
PD-1(+) CD4+ Cells, (%)	53.0	0.64	0.90	0.92	0.58	0.73	0.54
PD-1(+) CD8+ Cells, (%)	64.0	0.76	0.84	0.89	0.67	0.79	0.60
PD-1(+) CD3+ Cells + Child-Pugh score	0.80	0.71	1.0	1.0	0.66	0.81	0.71
PD-1(+) CD3+ Cells + CLIF-SOFA	0.65	0.80	0.8	0.88	0.69	0.80	0.60
PD-1(+) CD4+ Cells + Child-Pugh score	0.80	0.69	0.97	0.97	0.64	0.79	0.66
PD-1(+) CD4+ Cells + CLIF-SOFA	0.84	0.58	1.0	1.0	0.57	0.73	0.58
PD-1(+) CD8+ Cells + Child-Pugh score	0.81	0.71	1.0	1.0	0.66	0.81	0.71
PD-1(+) CD8+ Cells + CLIF-SOFA	0.79	0.71	1.0	1.0	0.65	0.81	0.71
Predicting 28-day mortality							
Child-Pugh score	11.0	0.81	0.66	0.75	0.74	0.74	0.47
CLIF-SOFA	10.0	0.79	0.68	0.76	0.71	0.74	0.47
PD-1(+) CD3+ Cells, (%)	62.0	0.65	0.79	0.79	0.64	0.71	0.44
PD-1(+) CD4+ Cells, (%)	54.0	0.65	0.82	0.82	0.65	0.72	0.46
PD-1(+) CD8+ Cells, (%)	65.0	0.77	0.74	0.79	0.72	0.76	0.51
PD-1(+) CD3+ Cells + Child-Pugh score	0.70	0.77	0.95	0.95	0.77	0.85	0.72
PD-1(+) CD3+ Cells + CLIF-SOFA	0.51	0.88	0.76	0.82	0.82	0.82	0.63
PD-1(+) CD4+ Cells + Child-Pugh score	0.70	0.73	0.92	0.92	0.73	0.81	0.65
PD-1(+) CD4+ Cells + CLIF-SOFA	0.66	0.69	0.92	0.92	0.69	0.79	0.61
PD-1(+) CD8+ Cells + Child-Pugh score	0.68	0.79	0.95	0.95	0.78	0.86	0.74
PD-1(+) CD8+ Cells + CLIF-SOFA	0.66	0.77	0.95	0.95	0.76	0.85	0.72

PPV, Positive predictive value; NPV, Negative predictive value; CLIF-SOFA, chronic liver failure-sequential organ failure assessment; Youden index =Sensitivity+Specificity−1. The value of the cut-off point is the one that maximizes the Youden index.

## Discussion

This study provides valuable insights into the prognostic role of lymphocyte PD-1 expression in patients with liver cirrhosis complicated by sepsis. The findings clearly demonstrate that higher PD-1 expression on CD3+, CD4+, and CD8+ lymphocytes is associated with worse clinical outcomes, including increased disease severity and 28-day mortality. This highlights the critical role of immune exhaustion in the pathophysiology of cirrhosis and sepsis, where impaired T-cell function and reduced pathogen clearance contribu1te to poor prognosis. These results are consistent with previous studies showing that excessive immune checkpoint expression, such as PD-1, is a hallmark of sepsis-related immunosuppression and is linked to higher rates of secondary infections and organ failure.

Severe sepsis is characterized by an initial cytokine-mediated hyperinflammatory response, followed by immunosuppression and secondary infections (13, 25). Compared to patients who die from non-sepsis-related causes, those who succumb to sepsis in the ICU exhibit biochemical, flow cytometric, and immunohistochemical changes consistent with immunosuppression (13, 26, 27). T-cell exhaustion is one of the key features of immunosuppression, and PD-1 serves as a critical inhibitory molecule in the activation of antigen-specific T cells (28). Previous studies have suggested that PD-1 expression on T lymphocytes can predict outcomes in septic patients (14, 28, 29). Furthermore, research has shown that patients with acute-on-chronic liver failure exhibit “sepsis-like” immune paralysis (15, 18). However, the relationship between T-cell PD-1 expression and disease severity or prognosis in patients with cirrhosis complicated by sepsis has not been fully explored (18). This study confirms this perspective by demonstrating that lymphocyte PD-1 expression is closely associated with disease severity and poor outcomes in this high-risk population.

The Child-Pugh score and CLIF-SOFA score are two widely used tools for evaluating disease severity and prognosis in patients with liver cirrhosis. The Child-Pugh score assesses liver function based on five parameters (bilirubin, albumin, prothrombin time, ascites, and hepatic encephalopathy), categorizing patients into three classes (A, B, and C), with higher scores indicating more severe liver dysfunction (23). The CLIF-SOFA score, on the other hand, evaluates multi-organ failure, including liver, kidney, respiratory, coagulation, and cardiovascular systems, making it more suitable for critically ill cirrhotic patients, particularly those with acute-on-chronic liver failure (ACLF) (19, 22). While the Child-Pugh score remains valuable for assessing long-term liver function and predicting complications like variceal bleeding (20, 23), the CLIF-SOFA score has been shown to better predict short-term mortality in critically ill patients, including those with cirrhosis complicated by sepsis. Studies have demonstrated that higher CLIF-SOFA scores are closely associated with increased mortality risk, making it an effective tool for monitoring disease progression and guiding clinical decisions (5, 19). Together, these scores offer complementary insights for risk stratification and prognosis in cirrhosis patients (30–38).

The positive correlation between PD-1 expression and clinical scoring systems, such as the Child-Pugh and CLIF-SOFA scores, further emphasizes the close relationship between immune dysfunction and disease severity. In particular, our findings suggest that PD-1 expression on CD8+ lymphocytes is associated with liver function deterioration, as reflected by the Child-Pugh score, while PD-1 expression on all lymphocyte subsets correlates with systemic organ failure, as indicated by the CLIF-SOFA score. These observations underscore the potential of PD-1 expression as a dynamic biomarker that reflects both hepatic decompensation and systemic dysfunction in patients with cirrhosis and sepsis.

Additionally, the multivariate logistic regression analysis confirmed that PD-1 expression on CD3+, CD4+, and CD8+ lymphocytes was an independent predictor of 28-day mortality. The integration of PD-1 expression with clinical scoring systems significantly improved the predictive accuracy for septic shock and mortality, as demonstrated by the ROC curve analysis. This combined approach offers a novel and more comprehensive method for risk stratification in this high-risk population. Clinicians could potentially use these findings to identify patients at higher risk early in their clinical course and tailor interventions accordingly.

From a clinical perspective, these findings also shed light on the potential utility of immune checkpoint inhibitors in treating sepsis-related immune dysfunction in cirrhotic patients. Previous studies have suggested that blocking the PD-1/PD-L1 pathway may restore T-cell function and improve immune responses in septic patients (11, 26). However, the safety and efficacy of such therapies in the context of liver cirrhosis remain to be fully explored. Given the delicate balance between immune activation and immune exhaustion in cirrhotic patients, careful patient selection and monitoring will be critical for the success of future interventions targeting PD-1.

Despite these promising results, the study has several limitations that warrant consideration. First, it was conducted at a single center with a relatively small sample size, which may limit the generalizability of the findings to broader populations. Second, although baseline biomarkers were associated with 28-day mortality, the follow-up period was relatively short, and we did not evaluate long-term outcomes; in future studies, we aim to conduct longer follow-up periods. Third, Our research only detected PD-1 levels on the surface of T cells and did not assess CTLA-4, LAG-1, and TIM-1 markers. Supplementing these markers would enhance the significance of the study. In the future, we plan to study larger cohorts of patients and dynamically monitor changes in these values for predictive purposes in patient prognosis.

In conclusion, this study demonstrates that lymphocyte PD-1 expression is a valuable biomarker for predicting disease severity and mortality in patients with liver cirrhosis complicated by sepsis. Combining PD-1 expression with traditional clinical scoring systems enhances prognostic accuracy and provides a more comprehensive approach to risk stratification. These findings not only deepen our understanding of immune dysfunction in this population but also open avenues for future research into immune-based therapies. Larger, multicenter studies are needed to validate these results and further explore the therapeutic potential of targeting PD-1 in cirrhosis and sepsis.

## Data Availability

The datasets used and/or analyzed during the current study are available from the corresponding author on reasonable request.

## Glossary

PD-1: Programmed cell death protein 1
CLIF-SOFA: chronic liver failure-sequential organ failure assessment
PBMCs: peripheral blood mononuclear cells
MAP: mean arterial pressure
COPD: chronic obstructive pulmonary disease
RR: respiratory rate
HR: heart rate
SBP: systolic blood pressure
PCT: Procalcitonin
CRP: C-reactive protein
HGB: hemoglobin
WBC: white blood cell count
NLR: neutrophil-to-lymphocyte ratio
INR: International normalized ratio
ALT: Alanine aminotransferase
AST: Aspartate aminotransferase
TBIL: Total bilirubin
DBIL: Direct bilirubin
PBS: phosphate-buffered saline
ROC: Receiver Operating Characteristic
ACLF: acute-on-chronic liver failure.
