# Supplementary material for: Prognostic significance of lymphocyte PD-1 expression in combination with clinical scoring systems in patients with liver cirrhosis complicated by sepsis
Source: Front Immunol. 2026 May 13;17:1749708. doi: 10.3389/fimmu.2026.1749708 (PMC13212349; doi:10.3389/fimmu.2026.1749708)
Supplement: Supplementary file 1 [file SupplementaryFile1.docx]

**Supplemental document**

**Table S1. Univariable and multivariable logistic regression analysis for the predictors of 28-day mortality by clinical factors and PD-1(+) CD3+ Cells**

| Characteristics | Total(N) | Univariate analysis | |  | Multivariate analysis | |
| --- | --- | --- | --- | --- | --- | --- |
|  |  | Odds Ratio (95% CI) | P value |  | Odds Ratio (95% CI) | P value |
| liver failure | 86 |  |  |  |  |  |
| 0 | 40 | Reference |  |  | Reference |  |
| 1 | 46 | 2.796 (1.161 – 6.736) | **0.022*** |  | 0.369 (0.042 – 3.285) | 0.372 |
| Hepatic encephalopathy | 86 |  |  |  |  |  |
| 0 | 33 | Reference |  |  | Reference |  |
| 1 | 53 | 5.827 (2.246 – 15.112) | **< 0.001*** |  | 0.826 (0.078 – 8.706) | 0.874 |
| Coronary heart disease | 86 |  |  |  |  |  |
| 0 | 61 | Reference |  |  | Reference |  |
| 1 | 25 | 4.714 (1.566 – 14.188) | **0.006*** |  | 5.589 (0.753 – 41.470) | 0.092 |
| COPD | 86 |  |  |  |  |  |
| 0 | 80 | Reference |  |  |  |  |
| 1 | 6 | 38492925 (0.000 – Inf) | 0.991 |  |  |  |
| Kidney failure | 86 |  |  |  |  |  |
| 1 | 57 | Reference |  |  | Reference |  |
| 0 | 29 | 0.093 (0.032 – 0.273) | **< 0.001*** |  | 0.115 (0.015 – 0.868) | **0.036*** |
| RR | 86 | 1.226 (1.066 – 1.411) | **0.004*** |  | 1.354 (1.000 – 1.833) | 0.050 |
| HR | 85 | 1.028 (1.005 – 1.052) | **0.019*** |  | 1.028 (0.970 – 1.089) | 0.358 |
| SBP | 86 | 0.967 (0.947 – 0.988) | **0.003*** |  | 0.994 (0.954 – 1.036) | 0.785 |
| Child-Pugh score | 86 | 1.641 (1.301 – 2.069) | **< 0.001*** |  | 1.792 (0.753 – 4.263) | 0.187 |
| CLIF-SOFA | 85 | 1.308 (1.152 – 1.486) | **< 0.001*** |  | 0.928 (0.661 – 1.302) | 0.664 |
| INR | 86 | 3.317 (1.452 – 7.574) | **0.004*** |  | 1.695 (0.352 – 8.147) | 0.510 |
| D-dimer | 85 | 1.000 (1.000 – 1.000) | 0.120 |  |  |  |
| Glucose | 79 | 0.909 (0.816 – 1.012) | 0.083 |  | 0.950 (0.783 – 1.154) | 0.606 |
| Lactic acid | 85 | 1.386 (1.143 – 1.681) | **< 0.001*** |  | 1.194 (0.818 – 1.743) | 0.359 |
| PD-1(+) CD3+ Cells, (%) | 86 | 1.092 (1.045 – 1.143) | **< 0.001*** |  | 1.144 (1.051 – 1.245) | **0.002*** |

Abbreviations: OR: odds ratio; 95% CI: 95% confidence interval. COPD, Chronic Obstructive Pulmonary Disease; RR, respiratory rate; HR, heart rate; SBP, systolic blood pressure; CLIF-SOFA, chronic liver failure-sequential organ failure assessment; INR, International normalized ratio. *p-value <0.05.

**Table S2. Univariable and multivariable logistic regression analysis for the predictors of 28-day mortality by clinical factors and PD-1(+) CD4+ Cells**

| Characteristics | Total(N) | Univariate analysis | |  | Multivariate analysis | |
| --- | --- | --- | --- | --- | --- | --- |
|  |  | Odds Ratio (95% CI) | P value |  | Odds Ratio (95% CI) | P value |
| liver failure | 86 |  |  |  |  |  |
| 0 | 40 | Reference |  |  | Reference |  |
| 1 | 46 | 2.796 (1.161 – 6.736) | **0.022*** |  | 0.353 (0.042 – 2.960) | 0.337 |
| Hepatic encephalopathy | 86 |  |  |  |  |  |
| 0 | 33 | Reference |  |  | Reference |  |
| 1 | 53 | 5.827 (2.246 – 15.112) | **< 0.001*** |  | 1.396 (0.145 – 13.451) | 0.773 |
| Coronary heart disease | 86 |  |  |  |  |  |
| 0 | 61 | Reference |  |  | Reference |  |
| 1 | 25 | 4.714 (1.566 – 14.188) | **0.006*** |  | 8.299 (1.056 – 65.202) | **0.044*** |
| COPD | 86 |  |  |  |  |  |
| 0 | 80 | Reference |  |  |  |  |
| 1 | 6 | 38492925 (0.000 – Inf) | 0.991 |  |  |  |
| Kidney failure | 86 |  |  |  |  |  |
| 1 | 57 | Reference |  |  | Reference |  |
| 0 | 29 | 0.093 (0.032 – 0.273) | **< 0.001*** |  | 0.173 (0.026 – 1.156) | 0.070 |
| RR | 86 | 1.226 (1.066 – 1.411) | **0.004*** |  | 1.309 (0.980 – 1.749) | 0.068 |
| HR | 85 | 1.028 (1.005 – 1.052) | **0.019*** |  | 1.031 (0.976 – 1.089) | 0.273 |
| SBP | 86 | 0.967 (0.947 – 0.988) | **0.003*** |  | 0.993 (0.953 – 1.035) | 0.746 |
| Child-Pugh score | 86 | 1.641 (1.301 – 2.069) | **< 0.001*** |  | 1.426 (0.660 – 3.084) | 0.367 |
| CLIF-SOFA | 85 | 1.308 (1.152 – 1.486) | **< 0.001*** |  | 0.980 (0.710 – 1.353) | 0.903 |
| INR | 86 | 3.317 (1.452 – 7.574) | **0.004*** |  | 1.884 (0.439 – 8.090) | 0.394 |
| D-dimer | 85 | 1.000 (1.000 – 1.000) | 0.120 |  |  |  |
| Glucose | 79 | 0.909 (0.816 – 1.012) | 0.083 |  | 0.930 (0.777 – 1.113) | 0.427 |
| Lactic acid | 85 | 1.386 (1.143 – 1.681) | **< 0.001*** |  | 1.196 (0.830 – 1.724) | 0.338 |
| PD-1(+) CD4+ Cells, (%) | 86 | 1.108 (1.051 – 1.167) | **< 0.001*** |  | 1.158 (1.052 – 1.274) | **0.003*** |

Abbreviations: OR: odds ratio; 95% CI: 95% confidence interval. COPD, Chronic Obstructive Pulmonary Disease; RR, respiratory rate; HR, heart rate; SBP, systolic blood pressure; CLIF-SOFA, chronic liver failure-sequential organ failure assessment; INR, International normalized ratio. *p-value <0.05.

**Table S3. Univariable and multivariable logistic regression analysis for the predictors of 28-day mortality by clinical factors and PD-1(+) CD8+ Cells**

| Characteristics | Total(N) | Univariate analysis | |  | Multivariate analysis | |
| --- | --- | --- | --- | --- | --- | --- |
|  |  | Odds Ratio (95% CI) | P value |  | Odds Ratio (95% CI) | P value |
| liver failure | 86 |  |  |  |  |  |
| 0 | 40 | Reference |  |  | Reference |  |
| 1 | 46 | 2.796 (1.161 – 6.736) | **0.022*** |  | 0.323 (0.035 – 2.962) | 0.317 |
| Hepatic encephalopathy | 86 |  |  |  |  |  |
| 0 | 33 | Reference |  |  | Reference |  |
| 1 | 53 | 5.827 (2.246 – 15.112) | **< 0.001*** |  | 0.878 (0.081 – 9.552) | 0.915 |
| Coronary heart disease | 86 |  |  |  |  |  |
| 0 | 61 | Reference |  |  | Reference |  |
| 1 | 25 | 4.714 (1.566 – 14.188) | **0.006*** |  | 7.014 (0.893 – 55.116) | 0.064 |
| COPD | 86 |  |  |  |  |  |
| 0 | 80 | Reference |  |  |  |  |
| 1 | 6 | 38492925 (0.000 – Inf) | 0.991 |  |  |  |
| Kidney failure | 86 |  |  |  |  |  |
| 1 | 57 | Reference |  |  | Reference |  |
| 0 | 29 | 0.093 (0.032 – 0.273) | **< 0.001*** |  | 0.113 (0.014 – 0.890) | **0.038*** |
| RR | 86 | 1.226 (1.066 – 1.411) | **0.004*** |  | 1.324 (0.969 – 1.809) | 0.078 |
| HR | 85 | 1.028 (1.005 – 1.052) | **0.019*** |  | 1.025 (0.967 – 1.086) | 0.414 |
| SBP | 86 | 0.967 (0.947 – 0.988) | **0.003*** |  | 0.993 (0.953 – 1.035) | 0.753 |
| Child-Pugh score | 86 | 1.641 (1.301 – 2.069) | **< 0.001*** |  | 1.598 (0.677 – 3.772) | 0.284 |
| CLIF-SOFA | 85 | 1.308 (1.152 – 1.486) | **< 0.001*** |  | 0.958 (0.682 – 1.346) | 0.806 |
| INR | 86 | 3.317 (1.452 – 7.574) | **0.004*** |  | 1.887 (0.380 – 9.367) | 0.437 |
| D-dimer | 85 | 1.000 (1.000 – 1.000) | 0.120 |  |  |  |
| Glucose | 79 | 0.909 (0.816 – 1.012) | 0.083 |  | 0.928 (0.772 – 1.117) | 0.432 |
| Lactic acid | 85 | 1.386 (1.143 – 1.681) | **< 0.001*** |  | 1.216 (0.799 – 1.848) | 0.361 |
| PD-1(+) CD8+ Cells, (%) | 86 | 1.113 (1.057 – 1.171) | **< 0.001*** |  | 1.160 (1.061 – 1.268) | **0.001*** |

Abbreviations: OR: odds ratio; 95% CI: 95% confidence interval. COPD, Chronic Obstructive Pulmonary Disease; RR, respiratory rate; HR, heart rate; SBP, systolic blood pressure; CLIF-SOFA, chronic liver failure-sequential organ failure assessment; INR, International normalized ratio. *p-value <0.05.


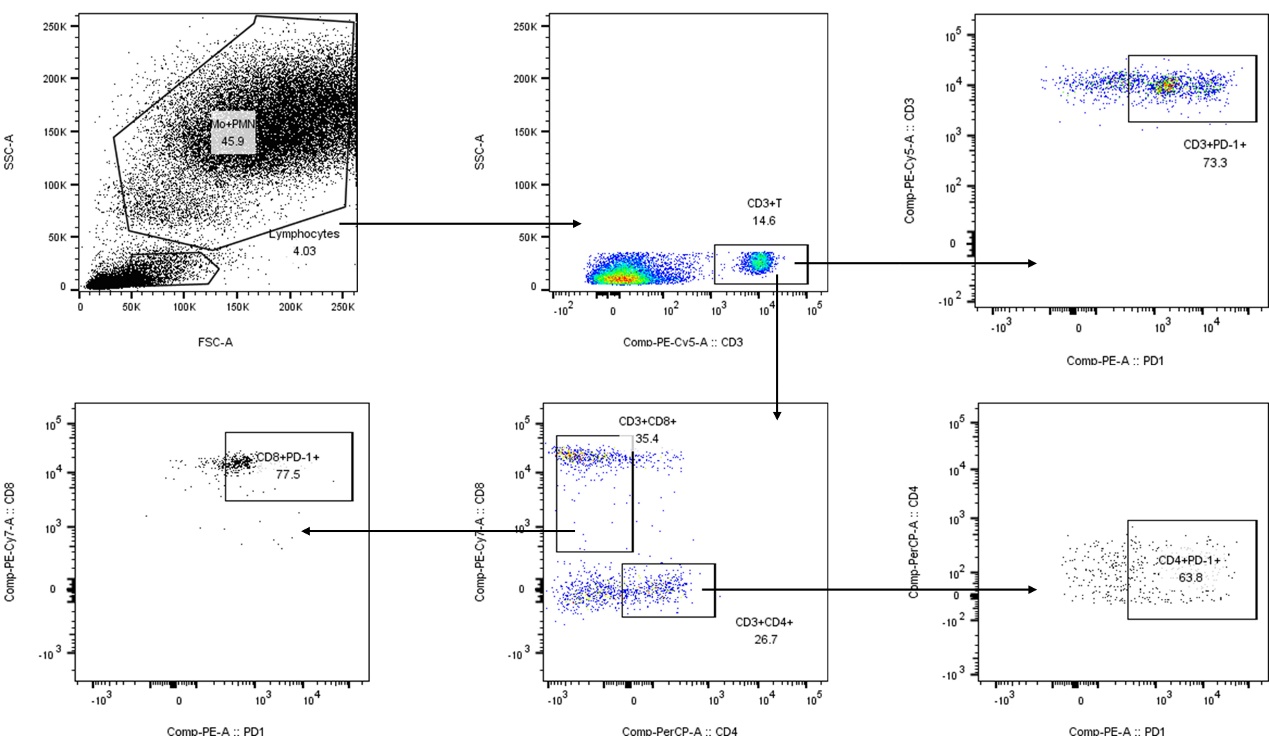


Figure S1. The process of flow cytometric analysis.


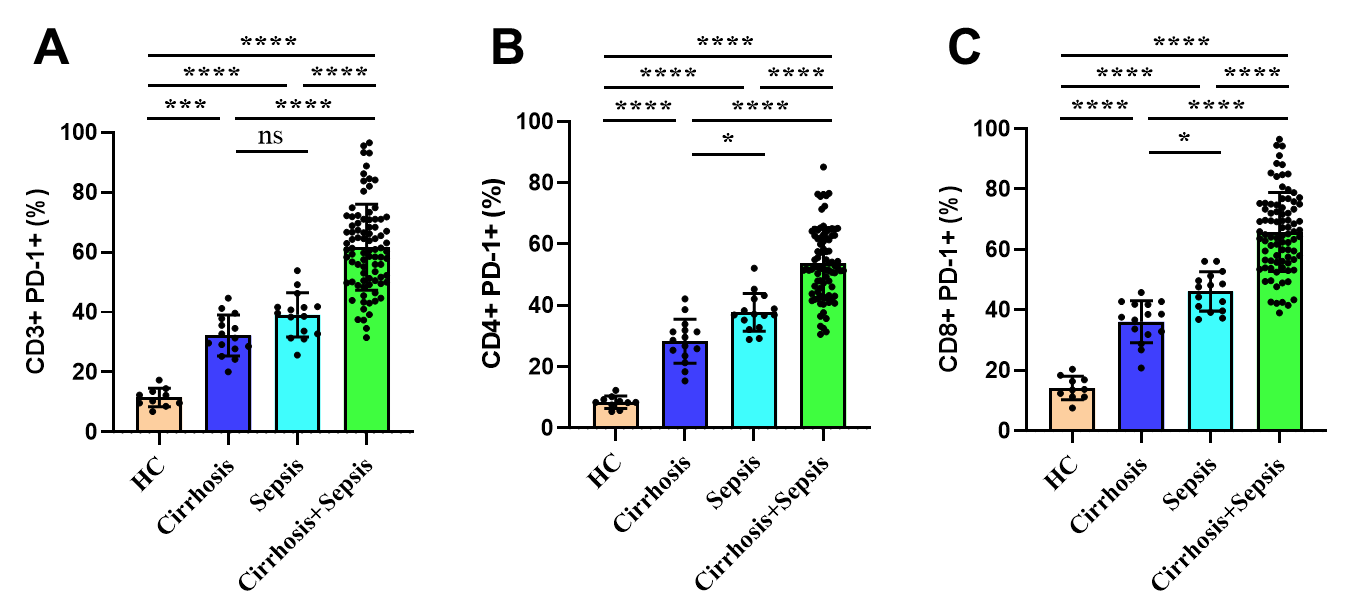


Figure S2. Lymphocyte PD-1 expression in different populations. This figure shows the proportion of CD3+PD-1+ cells (A), CD4+PD-1+ cells (B), and CD8+PD-1+ cells (C) in various populations. The data are presented as mean ± standard deviation (SD) and were compared using the Analysis of Variance (ANOVA). * indicates p < 0.05, ** indicates p < 0.01, and *** indicates p < 0.001.


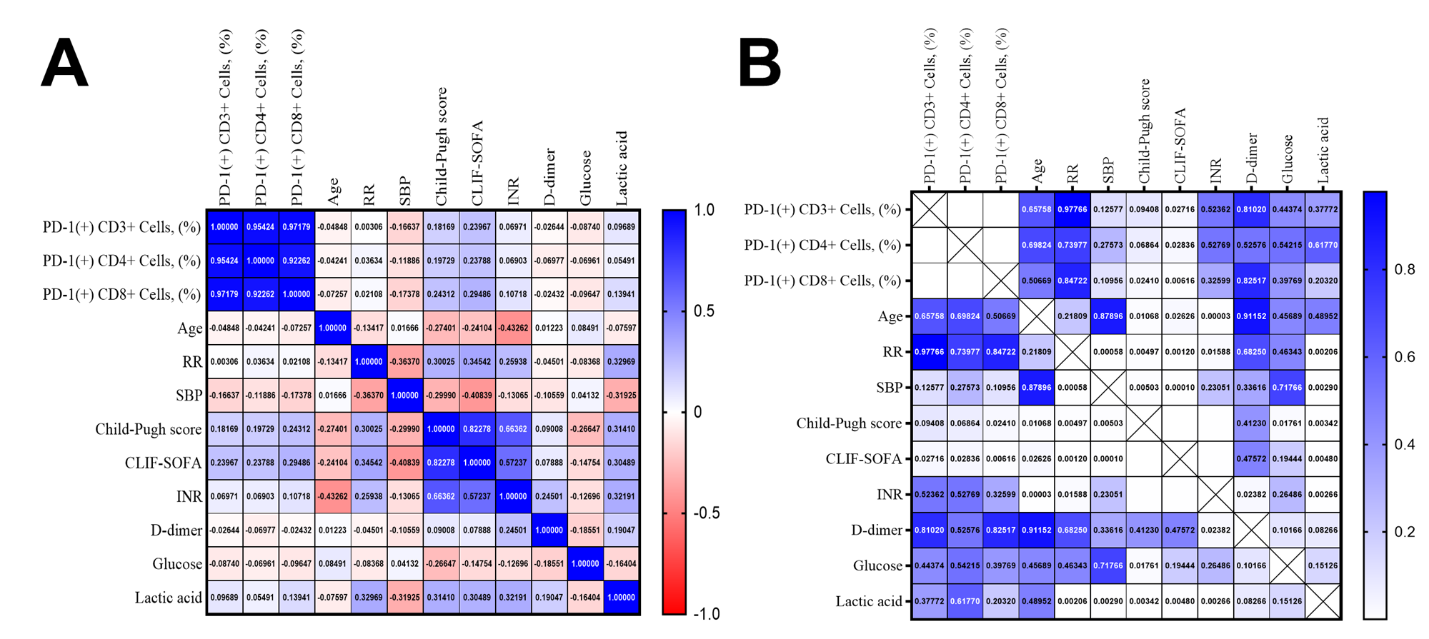


Figure S3. Heatmap depicting the correlation between Lymphocyte PD-1 Expression and age, Vital signs, and laboratory markers. (A) The values are presented as Spearman‘s correlation coefficient (r) for a sample of 86 runners. The colormap ranges from 1 to -1, with blue indicating the highest value and red indicating the lowest value. (B) The Heatmap of corresponding p-values. The colormap ranges from 0 to 1, with blue representing the largest value and white representing the smallest value. White cells without numerical values indicate that the p-value is smaller than 0.00001, indicating a highly significant correlation. Abbreviations: RR, respiratory rate; SBP, systolic blood pressure; CLIF-SOFA, chronic liver failure sequential organ failure assessment; INR, International normalized ratio.
